# Food Appreciation Scale Development and Dimensionality Assessment

**DOI:** 10.3390/ijerph20146345

**Published:** 2023-07-12

**Authors:** Kelly Cosgrove, Christopher Wharton

**Affiliations:** College of Health Solutions, Arizona State University, 550 N 3rd Street, Phoenix, AZ 85004, USA; christopher.wharton@asu.edu

**Keywords:** food appreciation, food waste, dietary habits

## Abstract

Food appreciation has been associated with favorable dietary and food waste behaviors. However, no validated food appreciation assessment currently exists. This study aimed to develop and validate a food appreciation scale (FAS) using data from two independent US-based samples recruited online. The 29-item FAS was based on existing literature regarding appreciation as a psychological construct, mindful eating, and epicurean tendencies. In Study 1, 311 participants completed the FAS, and exploratory factor analysis (EFA) was conducted. In Study 2, 300 participants completed the FAS, and confirmatory factor analysis (CFA) was conducted to determine whether the factor structure remained consistent. The EFA indicated a good model fit for a four-factor structure after excluding six items that loaded on multiple or no factors (TLI 0.95, CFI 0.97, SRMR 0.03, RMSEA 0.05), and Cronbach’s alpha indicated excellent reliability (Cronbach’s alpha values 0.82–0.9). The CFA confirmed the four-factor structure (TLI 0.97, CFI 0.98, SRMR 0.08, RMSEA 0.05) and acceptable factor loadings with a simple structure. The factors assessed active food appreciation, reflective food appreciation, mindful epicurean tendencies, and food-related rituals. The validated FAS could allow researchers to assess food appreciation, measure changes in food appreciation over time, and compare food appreciation among different study populations.

## 1. Introduction

Individuals’ connection to and relationship with food is complex and likely driven by a host of influential factors. How one enjoys or appreciates food might be affected by emotional states, sensory and aesthetic attributes of food, and notions about the social nature of producing and consuming food, among other factors [1,2]. As such, appreciation of food could potentially underlie a host of diet-related behaviors and health outcomes, including healthful dietary patterns, efficient use of food, overweight and obesity, and health-behavior-related risks, such as cardiovascular disease [1].

In response, a variety of methodologies orienting individuals towards eating with a heightened sense of enjoyment or appreciation have recently been developed and tested. Mindful eating, for example, represents an approach to eating that centers the experience and enjoyment of eating itself through heightened awareness of the process of eating without distraction [3]. Mindful eating has been shown in the past to be successful in some populations in terms of improving healthy eating practices and supporting weight management attempts [4]. A similar approach called intuitive eating orients individuals to internal versus external cues for choosing and eating foods in a healthy manner [1]. This approach has recently been shown to be associated with psychological benefits, such as perceived quality of life, and physical benefits, including improved body mass index (BMI) [5].

The connections between these types of intentional eating schemes and health and other outcomes require more research. However, the concept of food appreciation itself has a long history of being described in the literature as a food-related construct that is potentially associated with important dietary variables, such as food waste and diet quality [6,7,8]. Previous studies have indicated that food appreciation may act as a mediator of food waste behaviors as well as a predictor of dietary behaviors and health status [6,9]. A study investigating food waste in a school food service environment administered surveys to dietitians and nutrition teachers within the school [10]. The respondents reported that a lack of appreciation for food was a driver of plate waste among students. A review article discussing the causes of food waste and potential opportunities to decrease food waste also highlighted a lack of appreciation of food as a possible underlying factor contributing to food waste behaviors [11]. In addition to the possible link between food appreciation and food waste behaviors, gratitude for food has been suggested to decrease food dislikes in children, potentially leading to better diet quality [12]. As such, food appreciation, as a food-related construct, deserves further attention in the literature due to its associations with food waste and diet quality, as these are factors with important health and environmental consequences.

Food waste has been highlighted in the literature as an important driver of poor environmental outcomes and has been demonstrated to have important impacts on greenhouse gas emissions, energy use, food and water security, and land use [13,14,15,16]. Reducing food waste has become an important priority because of the associated negative financial and environmental consequences. Food waste occurs at every stage of food production and consumption processes, but food waste at the consumer level has started to receive attention as a potentially important target of interventions to reduce food waste. This strategy is likely important in developed countries, in particular, as the largest proportion of wasted food is at the consumer and retail levels. Additionally, specifically among households in industrialized countries, one of the largest categories of food waste is fruits and vegetables, making food waste a lost opportunity for eating healthfully and possibly impacting overall diet quality [17].

As another food-related behavior of interest, dietary intake has been widely accepted as a highly influential risk factor for many chronic diseases [18,19,20]. Better diet quality has been associated with a significantly lower risk of incident cardiovascular disease, which is a leading cause of death in developed countries [21]. Diet quality has also been linked to metabolic health more broadly, as diet quality has been identified as an independent factor influencing metabolic health, making diet quality an important target of health-promotion efforts [22].

Problematic, unintentional, or overall poor relationships with food could possibly lead to food being consumed and wasted in excess, contributing to the negative health and environmental outcomes associated with food waste and poor diet. Moreover, there is evidence that positive food-related psychosocial traits are associated with more favorable diet quality [23]. Improving consumers’ relationships with and attitudes toward food has the potential to improve both diet quality and related health outcomes, as well as food waste behaviors and related environmental outcomes. Although appreciation of food has been shown to be associated with food behaviors, to date, no measure of food appreciation has been validated. In previous studies, appreciation of food has been assessed using only unvalidated questions answered on a Likert scale. A validated measure of food appreciation would allow this potential driver of important food behaviors to be measured and compared among studies or included as an outcome of intervention studies. Therefore, in the current study, the Food Appreciation Scale (FAS) was developed, and its factor structure and dimensionality were assessed.

## 2. Study 1

Study 1 aimed to develop the FAS items, explore the scale’s factor structure, and assess the scale’s internal consistency reliability.

### 2.1. Methods

#### 2.1.1. Participants and Procedure

A total of 332 participants from Amazon Mechanical Turk (Mturk) were recruited using CloudResearch and completed a survey that was delivered online through Qualtrics. Previous studies have indicated that individuals residing in the U.S. who complete surveys on Mturk are more similar to the U.S. internet population and more diverse than traditional pools of subjects (e.g., college students) [24]. Moreover, a recent study was conducted in which the survey response quality of participants recruited through Mturk, Qualtrics, an undergraduate student sample, Prolific, or CloudResearch revealed that the participants recruited through Prolific or CloudResearch provided responses of higher quality, as assessed through attention checks, meaningfulness of answers, following of instructions, memory of previously presented information, unique IP addresses and geolocation, and pace of work that would allow participants to read all of the items [25]. Additionally, measures were taken to improve the quality of data obtained from this online sample. For example, attention check questions were included in the survey to ensure that the participants were reading the questions thoroughly and paying attention while answering the survey questions. The CloudResearch platform was also utilized to screen participants using the following criteria that have been widely used in previous research: 100+ human intelligence tasks (HITs) approved, approval rate >95%, duplicate IP block, suspicious geocode block, verify worker country location, and Cloud Research approved participants [26,27]. A recent study investigated the quality of data obtained from different research platforms and panels and revealed high data quality for samples recruited via CloudResearch using the above data quality filters [26]. The study was approved by the Institutional Review Board of Arizona State University (protocol code STUDY00015007), and all participants provided informed consent by reading and agreeing to the consent form provided through the Qualtrics survey prior to the completion of the survey and received USD 0.75 compensation for participating.

The median (interquartile range, IQR) age of the participants was 38 (31–50) years. The median annual income was USD 45,000 (USD 25,000–USD 65,000), and 45.9% of the participants were male. Participants were recruited using a convenience sample, which did not differ markedly from the general US population according to the 2020 and 2021 census data encompassing all US residents (median age 38.8 years, median annual income USD 69,717, and 49.5% male). All participants had complete data as the survey required all questions to be answered before moving to the next section.

#### 2.1.2. Food Appreciation Scale

Using a deductive method, items for the FAS were adapted from other scales assessing appreciation as a general psychological construct [28], mindful eating [29], and food enjoyment/epicurean tendencies [30]. Experts in the abovementioned areas, such as professors who are widely published in the area of appreciation as a psychological construct and researchers in the field of gratitude for food, reviewed the scale, and modifications were made to incorporate their feedback. For example, the response scale was modified to be more consistent and easier for participants to understand, and several items were reworded to improve clarity and increase the applicability of the items to people with different religious beliefs. The FAS contained 29 items that are rated on a seven-point Likert scale ranging from “strongly disagree” to “strongly agree” and can be found in the Supplementary Materials. The ratings provided on the FAS are averaged to obtain an overall score. Higher scores indicate a higher level of food appreciation.

#### 2.1.3. Statistical Analyses

The structure and dimensionality of the 29 items of the FAS were tested using an exploratory factor analysis (EFA) model, and Cronbach’s alpha was used to assess reliability. Data were screened for multivariate assumptions (normality, linearity, homogeneity, and homoscedasticity), and all assumptions were met. Twenty-one multivariate outliers were detected using Mahalanobis distance (X2(29) = 58.30), and they were removed from further analyses, resulting in a sample of 311 individuals. The EFA analysis was conducted using guidelines outlined by Preacher and MacCallum [31]. The level of significance was set to *p* < 0.05, and model fit was assessed according to the following cut-off values: comparative fit index >0.95, standardized root mean square residual <0.08, and root mean square error of approximation <0.06. All analyses were conducted using the psych package in R version 4.2.2.

### 2.2. Results

#### Exploratory Factor Analysis

Bartlett’s test indicated correlation adequacy (X2(406) = 5468.94, *p* < 0.001), and the KMO test indicated sampling adequacy (overall MSA = 0.93), suggesting that the data were a good fit for EFA. Parallel analysis and scree plot examination suggested four overall factors, whereas the Kaiser criterion suggested three overall factors. In the EFA models, direct oblimin rotation was used because of the expected factor correlation. A factor loading cutoff value of β ≥ 0.30 was adopted for the analyses. When testing all 29 questions with a three-factor model, two items (FAS4 and FAS6) did not load onto a factor, whereas one item (FAS22) loaded on two factors. Another three-factor model was tested after eliminating these three items, and the model achieved a simple structure, as all items loaded on one factor. This model had a moderate fit: RMSEA, 0.08; RMSR, 0.05; CFI, 0.89; and TLI, 0.85.

Next, a four-factor model was assessed. When testing all 29 questions with the four-factor model, one item (FAS4) did not load onto a factor, whereas three items (FAS3, FAS16, FAS22, FAS29) loaded on two factors according to a factor loading cutoff of β ≥ 0.30. Another four-factor model was tested after eliminating these five items, and the model achieved a simple structure, as all items loaded on one factor. The model also exhibited excellent fit: RMSEA, 0.05; RMSR, 0.03; CFI, 0.96; and TLI, 0.95. The 24-item four-factor model was retained, and the factor loadings are presented in Table 1.

Factor 1 included eight items that measured active appreciation of food with prompts such as “I am very thankful for the food I have” and “I appreciate the resources that went into producing the food I eat”. Factor 2 included six items that assessed reflection and thoughts about food with prompts such as “I often remind myself to be thankful for my food”. And, “I believe it is important to remind myself to be thankful for the food I eat on a consistent basis”. Factor 3 included seven items that appeared to assess present-moment appreciation and epicurean tendencies with prompts such as “Cooking is a major form of art, similar to music or painting” and “I often recognize and acknowledge how food makes me feel during and/or after a meal”. Finally, Factor 4 included three items that appeared to assess rituals related to food appreciation with prompts such as “I often give thanks for my food before I eat”. The reliability of all three factors was high, with Cronbach’s alpha values of 0.85, 0.90, 0.82, and 0.90 for Factors 1, 2, 3, and 4, respectively.

## 3. Study 2

In Study 2, confirmatory factor analysis (CFA) was conducted with a separate sample of participants to determine whether the factor structure and dimensionality remained consistent.

### 3.1. Methods

#### 3.1.1. Participants and Procedure

A total of 321 participants were recruited using the same methods described for Study 1, and participants in Study 2 completed the same survey administered through Qualtrics. The CloudResearch platform was used to exclude participants of Study 1 to ensure that an independent sample was recruited for Study 2. The study was approved by the Institutional Review Board of Arizona State University (protocol code STUDY00015007), and all participants provided informed consent by reading and agreeing to the consent form provided through the Qualtrics survey prior to the completion of the survey and received USD 0.75 compensation for participating.

The median (interquartile range, IQR) age of the participants was 37 (31–44.75) years. The median annual income was USD 49,500 (USD 28,125–USD 70,000), and 45.0% of the participants were male. All participants had complete data as the survey required all questions to be answered before moving to the next section.

#### 3.1.2. Statistical Analysis

R statistical software version 4.2.2 with the lavaan package was used to conduct the CFA using structural equation modelling. Data were screened for multivariate assumptions (normality, linearity, homogeneity, and homoscedasticity), and all assumptions were met. Additionally, there was no evidence of multicollinearity among the items (all variance inflation factors < 5). Twenty-one multivariate outliers were detected using Mahalanobis distance (χ2(29) = 58.30), and they were removed from further analyses, resulting in a sample of 300 individuals. There were no missing data because participants were required to complete all questions of the survey prior to submitting it. The level of significance was set to *p* < 0.05, and model fit was assessed according to the following cut-off values: comparative fit index >0.95, standardized root mean square residual <0.08, and root mean square error of approximation <0.06. The variances of the latent variables were fixed to 1 to set the scale of these latent variables, but all factor loadings, item variances, and factor covariances were estimated. Diagonally weighted least squares estimation was used for the model estimation.

### 3.2. Results

#### Confirmatory Factor Analysis

The sample size (N = 300) was sufficient for confirmatory factor analysis [32]. The four-factor model identified in the EFA exhibited excellent fit in this independent dataset (CFI = 0.977, RMSEA = 0.051, SRMR = 0.075).

**Figure 1:**
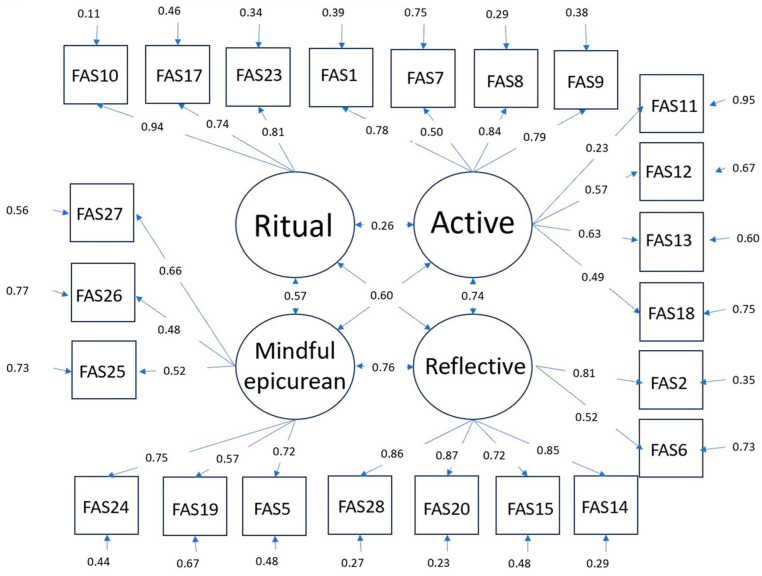
Path diagram of the four-factor model.

All of the factor loadings were >0.3 and significant (*p* < 0.01), with the exception of the factor loading of FAS11 (β = 0.22, *p* < 0.01). The CFA results indicated that the dimensionality and factor structure of the FAS remained consistent in a separate dataset. The path diagram with standardized estimates is shown in Figure 1. Unstandardized estimates are shown in Table 2.

## 4. Discussion

Although appreciation of food has been gaining more attention in the literature, a formal measure of food appreciation has not been developed to date. Food appreciation and gratitude have typically been assessed using unvalidated Likert-type scales, indicating the need for a validated measure of this potentially important food-related construct. In order to fill this gap and allow for comparable assessments of food appreciation, it is important that a food appreciation measure be developed and evaluated. In the current study, we developed a measure of food appreciation that initially included 29 items derived from the existing literature regarding appreciation as a general psychological construct [28], mindful eating [29], and food enjoyment/epicurean tendencies [30]. We then conducted two separate studies to assess the factor structure and dimensionality of the scale and determine whether the identified factor structure and dimensionality remained consistent in a separate sample of participants. The hypothesized structure determined in Study 1 was upheld in the CFA conducted in Study 2.

This newly developed FAS can be a useful tool for researchers aiming to assess appreciation of food in a way that is comparable between studies and multiple time points, for example, allowing for the assessment of food appreciation before and after an intervention targeting participants’ relationship with food. Moreover, the four factors identified in the FAS could provide information that could allow for more targeted interventions, such as those focused specifically on epicurean tendencies to increase one’s appreciation of food. A previous study indicated that the success of an intervention aimed at promoting healthier food choices depended upon the food-related values of the consumer, indicating the importance of assessing people’s relationship with and thoughts towards food [33].

Food appreciation is emerging in the literature as an important topic in a variety of fields. A study examined the appreciation of food authenticity in the context of local food production and found that personal identities and feelings toward local food production were important influencers of food choice [34]. A recent paper discussed the application of Allen Carlson’s object-centered model for the aesthetic appreciation of nature to food [35]. In this paper, the author stated that knowledge about the functions and history of food relate to food appreciation. A mixed-methods study was conducted to examine consumers’ perceptions of food waste, best before dates, and food appreciation in a supermarket in Germany [36]. The findings of the participant interviews related to food appreciation revealed that people felt that there was very little appreciation of food in Germany because of the abundance and availability of food, which has an important effect on food waste behaviors.

Other research in the area of food appreciation has examined the effects of sensory perceptions and psychological factors on the appreciation of food [37,38]. These studies have highlighted the complexity of decisions related to food behaviors. Much of the decision-making process occurs at the subconscious level and is influenced by psychological factors related to views of and relationships with food, along with food sensory-related factors and a multitude of non-food-related factors.

In this study, a scale was developed to assess the increasingly important construct of food appreciation, and the factor structure of the scale was assessed and confirmed in an independent sample. Despite the strengths of the study, certain limitations should be considered when interpreting the results. A limitation of this research is that an online convenience sample was utilized, which might limit the generalizability of the findings to other populations. In particular, this might be true when examining food appreciation among different cultures within the US as well as globally. However, online samples recruited using the CloudResearch platform have been shown to provide high-quality data and be more representative of the general population than other populations that are typically used for convenience sampling [26,27]. As described previously, efforts were made to increase the quality of data obtained from the CloudResearch sample. Despite these efforts to obtain high-quality data and the strengths of the CloudResearch platform, recruiting participants using CloudResearch might have led to selection bias. For example, because participants were recruited online to complete an online survey, it was necessary for them to have access to an internet-connected device, excluding the non-internet-user population. This could have resulted in a lack of representation of certain populations in the US, such as residents in rural regions who have more limited access to internet services than people who live in more urban areas [39]. Moreover, previous research has indicated that participant samples recruited using Mturk overrepresented younger, white, and male participants when compared to the demographics of the overall US population [40]. However, the demographic data from our sample indicate that the sample did not differ markedly from the general US population. It is still important that the FAS be evaluated in other populations, however. Future studies should use other recruitment methods with an option for non-digital survey completion to allow for the collection of data from a wider range of participants who might not have access to an internet-connected device or who prefer not to complete digital surveys.

The FAS items were developed based on theoretical speculations related to the appreciation of food. As this is a burgeoning area of research, there exists limited evidence regarding what constitutes appreciation of food. It is essential to conduct qualitative studies to expand this important area of research to ensure that all important facets of food appreciation are encompassed in the FAS.

Another important area of future research is to determine how the FAS score correlates with other measures and behaviors that would be expected to be related to one’s appreciation of food to indicate the validity of the FAS. Moreover, the temporal stability of the FAS should be explored.

## 5. Conclusions

In this study, a new scale measuring food appreciation was developed, and its dimensionality was assessed and validated. The scale demonstrated consistent factor structure in two independent samples of the US population. The scale is a promising tool to measure food appreciation in a variety of contexts, which can facilitate further research on the construct of food appreciation, which has been suggested as an important factor related to food waste and dietary behaviors. Future research should assess the validity of the scale in other populations and over time.

## Figures and Tables

**Table 1:** Four-factor model loadings.

Item	Factor 1	Factor 2	Factor 3	Factor 4
FAS1	**0.79**	−0.06	0.07	0.02
FAS7	**0.55**	0.01	−0.06	0.03
FAS8	**0.75**	0.06	0.04	0.09
FAS9	**0.60**	0.13	0.13	0.03
FAS11	**0.53**	0.04	−0.29	0
FAS12	**0.46**	0.20	0.10	−0.14
FAS13	**0.82**	−0.02	−0.01	−0.05
FAS18	**0.62**	0.17	0.02	−0.02
FAS2	0.04	**0.72**	0.02	0.10
FAS6	−0.19	**0.59**	0.27	−0.01
FAS14	0	**0.95**	−0.05	−0.02
FAS15	0.09	**0.74**	0.00	−0.07
FAS20	0.07	**0.78**	0.07	0.07
FAS28	0.2	**0.59**	0.02	0.18
FAS5	0.12	0.11	**0.52**	0.14
FAS19	0.21	0.05	**0.56**	−0.15
FAS21	0.06	0.18	**0.36**	0.17
FAS24	0.16	0.16	**0.47**	0.16
FAS25	−0.02	−0.11	**0.76**	0.07
FAS26	−0.10	0.07	**0.65**	−0.03
FAS27	0.06	0.07	**0.72**	0.03
FAS10	0.02	0.25	0.03	**0.65**
FAS17	−0.03	0.02	0.04	**0.85**
FAS23	0.01	−0.05	0.00	**0.99**

Note. Factor loadings have been sorted and bolded for ease of reading.

**Table 2:** Standardized and unstandardized coefficients.

Observed Variable	Latent Construct	β	B	SE
FAS1	Active	0.78	0.62	0.03
FAS7	Active	0.50	0.42	0.02
FAS8	Active	0.84	0.76	0.03
FAS9	Active	0.79	0.75	0.03
FAS11	Active	0.23	0.36	0.04
FAS12	Active	0.57	0.55	0.03
FAS13	Active	0.63	0.55	0.03
FAS18	Active	0.50	0.51	0.03
FAS2	Reflective	0.81	1.19	0.04
FAS6	Reflective	0.52	0.94	0.04
FAS14	Reflective	0.85	1.19	0.04
FAS15	Reflective	0.72	1.07	0.04
FAS20	Reflective	0.88	1.28	0.04
FAS28	Reflective	0.86	1.12	0.04
FAS5	Mindful epicurean	0.72	1.06	0.05
FAS19	Mindful epicurean	0.58	0.71	0.04
FAS24	Mindful epicurean	0.75	1.08	0.05
FAS25	Mindful epicurean	0.52	0.95	0.05
FAS26	Mindful epicurean	0.48	0.76	0.04
FAS27	Ritual	0.66	0.95	0.04
FAS10	Ritual	0.95	1.83	0.07
FAS17	Ritual	0.74	1.49	0.06
FAS23	Ritual	0.81	1.67	0.07

## Data Availability

The data presented in this study are available from the corresponding author upon reasonable request.

## Supplementary Materials

The following supporting information can be downloaded at: https://www.mdpi.com/article/10.3390/ijerph20146345/s1. Food appreciation scale.
