# Supplementary material for: Food Appreciation Scale Development and Dimensionality Assessment
Source: Int J Environ Res Public Health. 2023 Jul 12;20(14):6345. doi: 10.3390/ijerph20146345 (PMC10378760; doi:10.3390/ijerph20146345)
Supplement: Supplementary file 1 [file ijerph-20-06345-s001.zip › ijerph-2454970-Supplementary Materials.pdf]

Food appreciation scale

All items are scored on a 7-point scale from ‘strongly disagree’ to ‘strongly agree’.

1. I am very thankful for the food I have.
2. I often remind myself how fortunate I am to have access to food.
3. I often reflect on how fortunate I am to have food.
4. It is important to be thankful for the food I have access to.
5. I often give thanks for my food before I eat.
6. I often remind myself to be thankful for my food.
7. I often perform rituals around food (i.e., give thanks or say grace before a meal)
8. I often use personal or religious rituals to remind myself to be thankful for the food I eat.
9. I believe it is important to remind myself to be thankful for the food I eat on a consistent basis.
10. I often enjoy the experience of eating.
11. I often eat food while doing other tasks (e.g., read, watch TV, use phone).
12. I often notice the texture of my food while I’m eating.
13. I often recognize and acknowledge how food makes me feel during and/or after a meal.
14. I am usually fully present when I eat my food
15. I often pay attention only to the experience of eating.
16. I place special, positive meaning to regular meals that I often eat.
17. As I am eating, I think of times when I have been hungry and realize how fortunate I am to have food at the moment.
18. I often think of people who do not have access to food to feel more grateful for the food I have.
19. When I see or think of someone who does not have access to food, I realize how lucky I am.
20. I say “please” and “thank you” when people serve me food.
21. I recognize the human labor required for the food I eat.
22. Food is a basic need that I do not need to be grateful for because I am entitled to it.
23. I am very fortunate for the variety of food that I have access to.
24. I appreciate the resources that went into producing the food I eat.
25. There is a lot of beauty in food.
26. More than other people, I value the look, the smell, the taste, and the texture of foods.
27. If I try, I can clearly and easily imagine the taste of many dishes.
28. My friends say that I am a foodie.
29. Cooking is a major form of art, similar to music or painting.
